# Platelet Rich Plasma and Knee Surgery

**DOI:** 10.1155/2014/890630

**Published:** 2014-09-02

**Authors:** Mikel Sánchez, Diego Delgado, Pello Sánchez, Nicolás Fiz, Juan Azofra, Gorka Orive, Eduardo Anitua, Sabino Padilla

**Affiliations:** ^1^Arthroscopic Surgery Unit, Hospital Vithas San Jose, C/Beato Tomás de Zumarraga 10, 01008 Vitoria-Gasteiz, Spain; ^2^Arthroscopic Surgery Unit Research, Hospital Vithas San Jose, C/Beato Tomás de Zumarraga 10, 01008 Vitoria-Gasteiz, Spain; ^3^Fundación Eduardo Anitua, C/Jose María Cagigal 19, 01007 Vitoria-Gasteiz, Spain

## Abstract

In orthopaedic surgery and sports medicine, the knee joint has traditionally been considered the workhorse. The reconstruction of every damaged element in this joint is crucial in achieving the surgeon's goal to restore the knee function and prevent degeneration towards osteoarthritis. In the last fifteen years, the field of regenerative medicine is witnessing a boost of autologous blood-derived platelet rich plasma products (PRPs) application to effectively mimic and accelerate the tissue healing process. The scientific rationale behind PRPs is the delivery of growth factors, cytokines, and adhesive proteins present in platelets and plasma, as well as other biologically active proteins conveyed by the plasma such as fibrinogen, prothrombin, and fibronectin; with this biological engineering approach, new perspectives in knee surgery were opened. This work describes the use of PRP to construct and repair every single anatomical structure involved in knee surgery, detailing the process conducted in ligament, meniscal, and chondral surgery.

## 1. Introduction

**Figure 1:**
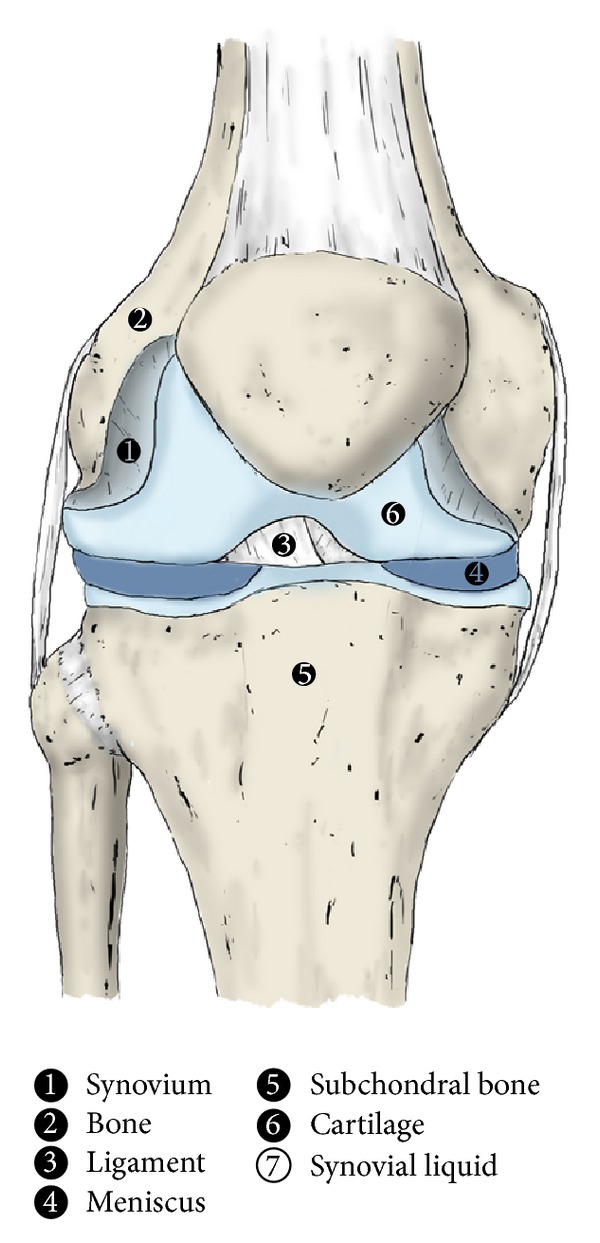
Anatomical structures of knee joint. The complexity and integration of all the structures that compose the knee joint make this have to be considered as an organ. Those structures are synovium (1), bone (2), ligament (3), meniscus (4), subchondral bone (5), cartilage (6), and synovial fluid (7), which coat the joint.

In orthopaedic surgery and sports medicine, the knee joint has traditionally been considered the workhorse. Unlike other synovial joints of the body, the knee encompasses a cluster of anatomical structures such as meniscus, extra- and intra-articular ligaments, bones, cartilage, and periarticular muscles; its integrity, congruity, and alignment guarantee its dynamic yet fragile stability, as well as its capacity to face extremely demanding biomechanical tradeoffs (Figure 1). From a mechanical viewpoint, the knee is a complex, shock-absorbing interface in which a coordinated and sequentially ordered engagement of the joint's elements and muscles is required to maintain the physical integrity of anatomical structures and homeostasis of knee tissues. In this respect, proprioceptive acuity, which depends on periarticular knee tissues, will play a crucial role in both knee injury mechanisms and rehabilitation. From a biomechanical perspective of the knee joint, it is most efficacious to present a holistic approach when the intention is to restore the function of such fragile joint [1]. Grasping the significance of the interplay among the aforementioned knee structures and knee function, it is easy to recognize that the reconstruction of every damaged element in this joint will be crucial in achieving the surgeon's goal to restore the knee function and prevent degeneration towards osteoarthritis. Both intense physical activity, a common feature shared by elite sports, and the absence of surgical repair chiefly of ACL injury that gives rise to act the knee joint as an eccentric structure are likely to result in a joint degenerative osteoarthritis [2, 3]. In the last fifteen years, the field of regenerative medicine is witnessing a boost of autologous blood-derived platelet rich plasma products (PRPs) application to effectively mimic and accelerate the tissue healing process [4].

Ten years ago, Sánchez et al. [5, 6] published two important papers depicting the application of plasma rich in growth factor (PRGF-Endoret) on arthroscopic surgery of the knee, being the first to report the successful application of PRGF-assisted regenerative techniques in the treatment of an articular cartilage avulsion in a 12-year-old soccer player [5]. With this biological engineering approach, new perspectives in knee surgery were opened. Furthermore, Sánchez et al. [5, 6] pioneered the comprehensive distributed application of PRGF-Endoret at different surgical junctures in the ACL reconstruction process, drawing on the paradigm of tissue-engineering biology. This procedure involves the use of activated liquid PRGF-Endoret to infiltrate the allografts or autografts achieving its biological reconditioning, to immerse bone plugs, and to fill the tibial and femoral tunnels in the operating theater [6]. Since then, several groups have not stopped harnessing the paradigm of tissue-engineering biology to construct and repair every single anatomical structure of the musculoskeletal system, including tendons, bones, cartilage, muscles, meniscus, and ligaments [7–12]. Despite the care and seriousness with which the medical staff elaborate and apply PRPs in different medical fields, the poor standardization in PRP therapies, the modalities of their application, and the* in vitro* versus* in vivo* assessments are elements that somehow are hampering advancement as well as drawing misleading conclusions about their clinical efficacy. It is now commonplace to apply PRPs or even unguided injections of autologous whole blood to manage musculoskeletal injuries as a magic bullet instead of adopting a rationale evidence based biological approach. In the wake of poor clinical results shown by this approach, it is tempting to make inferences about other autologous platelet rich plasma products, thereby suggesting that all these blood-derived products are useless in the treatment of, for example, tendinopathies. The approach of using PRGF-Endoret tissue-engineering biology, both* in situ* and in* operating theater*, has yielded extremely promising outcomes in the treatment of musculoskeletal system pathologies [9, 13–18].

## 2. Highlighting Features of the Use of PRP Products in Knee Surgery

**Figure 2:**
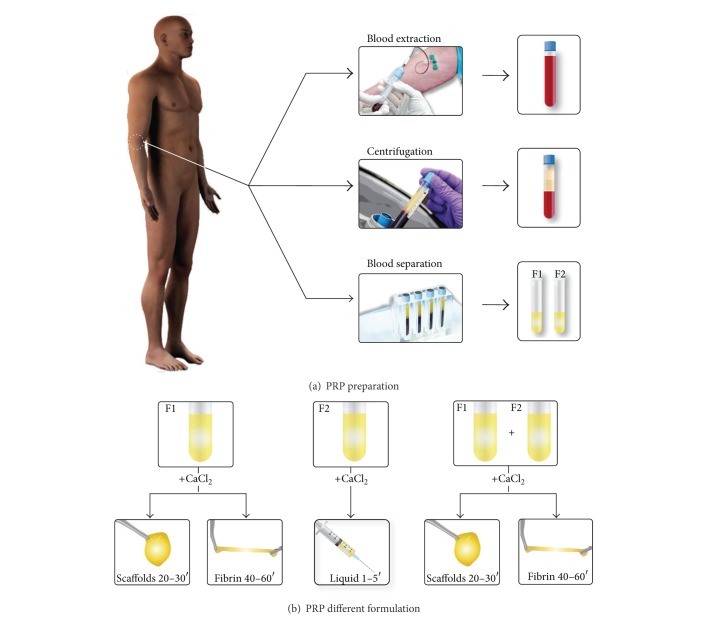
Platelet rich plasma protocol. Obtaining platelet rich plasma involves the extraction of a small volume of blood from the patient, its centrifugation to fractionate the blood, and the separation of platelet rich fractions (F1 and F2) (a). After activation of PRP fractions with calcium chloride, various formulations including liquid, clot, and membrane (b) can be obtained.

The scientific rationale behind PRPs is the delivery of growth factors, cytokines, and adhesive proteins present in platelets and plasma, as well as other biologically active proteins conveyed by the plasma such as fibrinogen, prothrombin, and fibronectin [19]. PRGF-Endoret is an autologous enriched platelet plasma product that does not contain leukocytes and is obtained by spinning a small sample of a patient's blood using a defined protocol [20] (Figure 2). After centrifugation, an autologous “liquid formulation” based on platelet enriched plasma is obtained. From this initial formulation we can prepare four distinct therapeutic formulations by adding calcium chloride to this liquid, thereby unleashing the activation of platelets and the polymerization of fibrin [13]. In orthopaedic surgery we primarily use an activated liquid formulation, a fibrin scaffold, or a fibrin membrane. They are all biomaterials conceived and prepared* in situ*, a procedure which follows a kind of Occam's razor: making biological engineering simple can be best achieved attempting to keep it simple [21] by applying nature's original technology. The technology of PRGF-Endoret mimics and harnesses the spontaneous defense-repair mechanisms with two biological outcomes: it avoids the formation of scar tissue which might lead to the loss of functionality and shortens the duration of repair events. The effectiveness of the application of PRGF-Endoret on knee surgery includes its mediation by multiple soluble biomolecules which are locally conveyed, namely, growth factors and cytokines, and stem from the activated platelets, plasma, and three-dimensional fibrin network.

Some growth factors present in platelets such as PDGF and TGF*β* have been shown to promote the proliferation of osteoblasts [22]. PRP preparations facilitate bone repair* in vitro* and* in vivo* by expressing the proosteogenic and angiogenic functions of endothelial cells, recruiting osteoblast precursors, and the expression of adhesion molecules (osteoprotegerin) while inhibiting their proosteolytic activity [23]. In two recent clinical studies, Seijas's and our group have shown that the application of PRGF-Endoret in the treatment of nonunion and delayed consolidation fractures might be both osteoinductive and osteoconductive [24, 25] by means of chemotaxis and osteoblast activity through growth factors such as PDGF, IGF-I, and TGF*β*1 [26]. This research evidence supports the application of activated liquid PRGF-Endoret in bone regeneration of the tibial and femoral tunnel in ACL reconstruction.

Of further interest, there is a great deal of evidence illustrating the anabolic effects of PRPs on tendon cells. PRPs stimulate the synthesis of types I and III collagen and cartilage oligomeric matrix proteins, resulting in a synthesis of extracellular matrix which is conducive to the osseointegration of grafts [11, 26, 27]. The wide spectrum of* in vitro* and* in vivo* cell response in both tendon stem cell differentiation and proliferation, together with a substantial expression of VEGF and HGF that generates a balanced angiogenesis and an anti-inflammatory effect, constitutes the rationale for the application of activated liquid and fibrin scaffolds; they are applied in the donor site of the graft to prompt the repair events in one area with a great deal of morbidity [12, 27–31]. The infiltration of activated liquid PRGF-Endoret to a previous implantation in Hamstring tendon graft elicits a set of sequential remodeling events that leads to the ligamentization of the tendon graft [11]. In one study conducted by our group we compared the overall arthroscopic evaluation and found the morphology and histology of tendon grafts treated with infiltration with more signs of remodeling, maturation, and a synthesis of new connective tissue than the nontreated one; moreover, the infiltrated tendon graft presented more and better-oriented cells and more akin to the native ACL [11]. A key aspect to consider is the TGF-*β*1 family, which drives fibrogenesis and potentially might stimulate the formation of scar tissue in the tendon graft; however, the fibrotic effect of TGF-*β*1 present in PRGF-Endoret would be either modulated, counterbalanced, or even hindered by the presence and local production of VEGF and HGF, a potent antifibrotic and anti-inflammatory agent [32, 33], as has been shown by our work on cells cultured on fibrin matrices [27, 34, 35]. Therefore, the concurrent presence of TGF-*β*1, VEGF, and HGF in the same local environment makes the PRGF-Endoret an antifibrotic and antiapoptotic autologous system and a useful toolkit for contributing to musculoskeletal tissue repair [36].

Cartilage is another knee structure often damaged and difficult to repair that can benefit from the healing potential of PRGF-Endoret. Growth factors conveyed by platelet rich plasma have been shown to produce a chondroprotective effect in the synovial joint due to the hyaluronic acid secretion by synoviocytes [37]. In addition, type II collagen cleavage can be arrested by the presence of TGF*β* and FGF and thereby contribute to the homeostasis of articular cartilage [38, 39]. Last but not least, PRGF-Endoret has been revealed as a mighty anti-inflammatory response that might be mediated on the basis of the high concentration of HGF present in PRP, besides being secreted by several cells, thereby inhibiting the intracellular signaling regulator of the inflammatory and stress-induced response pathway NF-k*β*  [32, 40].

## 3. PRP and Ligament Injuries

A ligament is a fibrous connective tissue band that connects bones together and is essential for joint stability. This is achieved by its mechanical behavior and its viscoelastic composition, which prevents the excessive motion caused by different forces exerted on the joint. Ligaments are composed of 70% water and 30% solid material, mainly extracellular matrix (EMC) (80%) and fibroblasts (20%), the most abundant cell elements in this anatomical structure. Concerning EMC, collagen is the most characteristic protein of the ligament reaching 75% of the dry weight and is divided into collagen fibers type I (90%) and type III (10%) [41]. These collagen fibers are arranged in a wide variety of directions and orientations since ligaments are submitted to several torsion and traction forces. Other extracellular matrix proteins present in ligaments are proteoglycans, elastin, actin, laminins, and integrins. The entire ECM is formed by fibroblasts, which are also responsible for the maintenance and repair of this tissue [42].

Ligaments are covered by the epiligament, which provides the microvascularity, and proprioceptive and nociceptive nerve endings, by which the organism is able to involuntarily detect the position and the movement of the knee. However, this vascular contribution is limited in the ligaments, a tissue with scant regeneration properties. This condition hampers its recovery from injuries and favors relapses.

There are different ligaments present in the knee; however we will use the anterior cruciate ligament (ACL) to exemplify the use of PRGF-Endoret together with the surgical reconstruction procedure. The ACL is frequently damaged in the field of sports and its poor repair ability causes the joint to work in an eccentric manner, triggering early knee osteoarthritis and making its surgical reconstruction almost mandatory [3]. Before explaining this technology, it is necessary to understand the regeneration process which takes place in ligament injury. In this way, PRGF-Endoret technology will be better understood and used in these types of procedures.

### 3.1. Ligament Repair Process

When ligament rupture occurs due to excessive mechanical energy, the vascular elements and extracellular matrix are disrupted. Consequently, there is an extravasation of plasma and blood cells into the damaged area and into the surrounding tissues [43]. Then, the mesenchymal stem cells are activated and migrate from their niches to the injured site [44]. Both these stem cells and endothelial and blood cells (platelets and macrophages) release growth factors and cytokines causing heat, edema, pain, and dysfunction in order to protect the knee from further damage. Cytokines attract macrophages and monocytes that remove those proteins and cells remaining in the damaged area, which is filled with plasma elements and blood cells. In addition, a fibrin clot is formed to integrate platelet and mesenchymal stem cells, which release molecules involved in repair processes. Simultaneously with activation and cell migration, angiogenesis occurs, and thereby new blood vessels are created and new extracellular matrix is synthesized [44]. Furthermore, the fibrin clot and its environment begin to transform into granular scar tissue where fibroblasts synthesize collagen types I and III, among other proteins. Finally, the remodeling process begins, which is a long stage characterized by a drop in cellularity, vascularity, and water content.

Bearing this in mind the use of PRP in ACL reconstruction using hamstring autografts, a standard technique in this condition, can be better understood. Although it often produces good results, this technique also suffers from considerable variability in both final outcomes and recovery time [45]. For this reason, ACL reconstruction is under constant revisions in which aspects such as graft type, position of the tunnels, or anchor methods are studied.

However, the biological aspect must not be ignored. In ACL reconstruction [11] PRGF-Endoret induces the proliferation of cells in tendon used as graft. Angiogenesis is promoted, accelerating the processes of remodeling, ligamentization, and integration of the graft. Together with an adequate physiotherapy that generates appropriate mechanical stimuli [46], this biological intervention achieves better and faster recovery of the patient who is undergoing this surgical procedure [6].

### 3.2. Arthroscopic Anterior Cruciate Ligament Reconstruction Associated with PRGF-Endoret

The following profile describes the process of ACL reconstruction by arthroscopy combined with PRGF-Endoret and using the autografts of semitendinosus tendon, gracilis tendon, and bone-tendon-bone patellar tendon [12].

**Figure 3:**
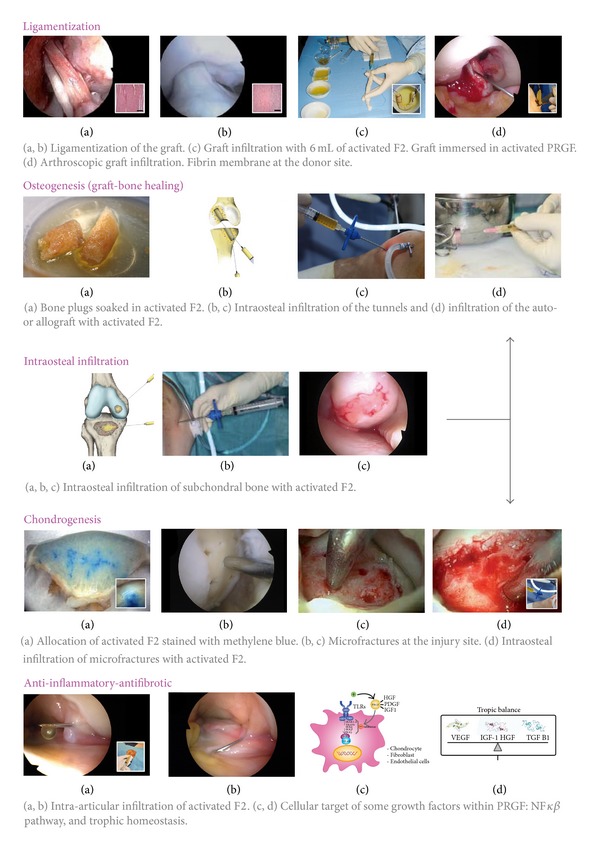
Platelet rich plasma and knee surgery. Platelet rich plasma can help in different surgical processes of the knee due to its effects on ligamentization, osteogenesis, or chondrogenesis as well as its anti-inflammatory and antifibrotic properties.

1. Before inducing anesthesia, prophylactic antibiotic treatment, and saline, seventy-two mL of peripheral venous blood is withdrawn into 9 mL tubes containing 3.8% (wt/vol) sodium citrate as anticoagulant. Blood is centrifuged at 580 g for 8 minutes at room temperature (PRGF-Endoret, Vitoria, Spain) (Figure 2). The upper volume of plasma contains a similar number of platelet as peripheral blood, and it is drawn off and deposited in a collection tube (F1). The 2 mL plasma fraction, located just above the sedimented red blood cells, is collected in another tube without aspirating the buffy coat. This plasma contains a moderate enrichment in platelets (2-3-fold the platelet count of peripheral blood) with scarce leukocytes (F2).
2. F1 is activated with calcium chloride (10% wt/vol) and incubated at 37°C for 30–60 minutes in a glass dish, to allow the formation of either a biocompatible fibrin scaffold or a fibrin membrane that will be placed in the donor region of the goose's foot tendon at the end of the process. In the case of using a bone-tendon-bone autograft, a fibrin scaffold is placed in the area where the graft was obtained, namely, tibia, patella, and patellar tendon; in addition, F2 activated with calcium chloride will be infiltrated in an intraosseous manner.
3. An assessment of the joint is conducted by arthroscopy in order to detect associated pathologies such as meniscal, synovial, or chondral injuries.
4. Once the remains of the ACL are cleaned, a condyloplasty is initiated to prevent future graft impingements, especially in chronic cases with narrower groove, and to promote the correct location of the femoral anchor point of the graft. It also will create a bed of bleeding spongy bone, providing cells and proteins that will enhance the integration of the graft.
5. When the joint site is prepared, the autologous grafts are obtained from those places already indicated. If allografts are used, they will have been prepared beforehand. Calcium chloride is added to the F2 aliquots just before infiltration; then, six milliliters of activated F2 is injected within the tendinous fascia of graft fascicles (auto- or allografts) in the operating theater itself (10 mL syringes and 21 G needles). The graft is immersed in a recipient with activated F2 until implantation.
6. The tunnels are produced using the selected procedures and guides. As in our surgical technique, it is important that the guide allows bone plugs to be extracted. These plugs are soaked in activated F2 and when the graft (in the case of goose's foot tendons) has been introduced, they are reimplanted; thus, the tibial tunnel is sealed supplying a biological anchorage.
7. As PRP can be removed by irrigation saline, the inlet is closed when the graft is placed. Furthermore, the remaining saline is aspirated to prevent dilution of PRP.
8. Three mL of F2 is injected with long needles into each bone tunnel after graft fixation. In this way the bone is exposed to a source of proteins and cells that enhance graft integration. After placing the graft in the tunnels, it is again infiltrated with activated F2, since some of the previously infiltrated PRP may be lost during this process. Finally, an intra-articular infiltration is carried out with the remaining F2 (Figure 3).

To complete the whole of the procedure it is necessary to implement mechanical stimuli by means of a rehabilitation plan and physiotherapy. The achieved mechanotransduction stimulates the cells in order to act synergistically with this surgical technique and PRP [46].

The proper execution of this process (surgery, PRP, and rehabilitation) will improve patient recovery. It can be seen postoperatively by a decrease in the number of hematomas and signs of inflammation such as pain. There is also a better osseointegration of the graft and as a result a better adaptation in the joint kinematics. All this leads to a shortening of the time of initiation of rehabilitation [6].

Posterior cruciate ligament, medial collateral ligament, and lateral collateral ligament can be reconstructed by applying the same principles described for ACL reconstruction, both arthroscopically and via open surgery.

## 4. PRP and Meniscal Surgery

The meniscus is an intra-articular structure formed by fibrocartilaginous tissue, composed mainly of type I collagen fibers (more than 90%), and is frequently damaged, affecting the knee stability and lubrication. Meniscus reparation process is determined by its tissue characteristics such as its abundant extracellular matrix (between 60 and 70% of tissue weight) where cells, namely, fibrochondrocytes, fibroblasts, and cells of the surface area, are dispersed. Moreover its poor vascularity is limited to 10–30% of its outer portion or meniscal wall, which also receives nerve endings and presents the most cellularity [47]. Such zone differentiation conditions meniscus recovery capacity, a decisive factor if the injury occurs in the central area or in the peripheral area (meniscal wall), which is the reparative part and generates regeneration processes [48].

Meniscus injuries compromise joint functions, as this structure provides stability to the knee and supports compressive stress as well as traction and shearing forces. Meniscus also absorbs some of the mechanical stress that the joint receives and participates in the lubrication of the knee with synovial membrane. Because of its functional importance in the knee and its vulnerability to repetitive injures throughout the lifetime of a person, it is necessary to improve its limited reparative capacity to achieve an optimal recovery.

In laboratory experiments, PRP has proved to have a positive effect on meniscal cells [49] and it has been proposed as a treatment for meniscal tears [50]. However, to use PRPs properly, it must be applied following the correct protocol appropriate indications. In surgical procedures, its use is focused on meniscectomies and meniscal sutures, by applying the activated F2 especially in the area of the meniscal wall.

### 4.1. Meniscectomy

**Figure 4:**
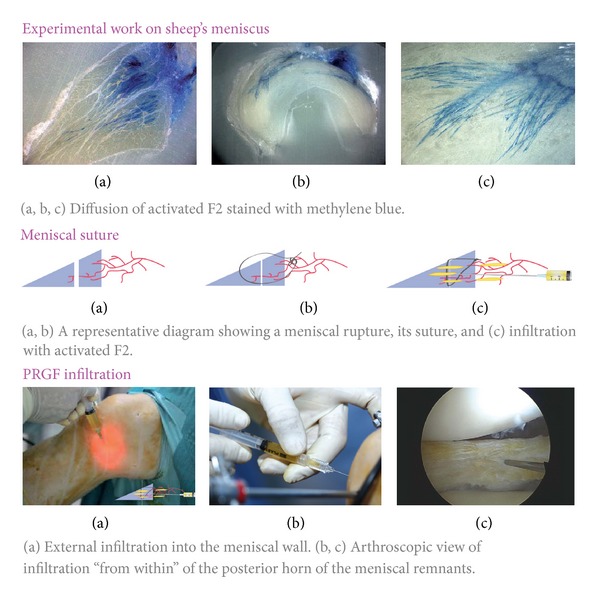
Platelet rich plasma and meniscus. Factors such as the spread of platelet rich plasma in the meniscus and tissue density require a proper protocol in order to perform a correct application.

As mentioned above, the meniscal wall is infiltrated with activated F2 during a partial or subtotal meniscectomy. This infiltration is carried out in an extra-articular way (from outside to inside) using a 21 G needle and a 3 mL syringe. An exception to this procedure is the infiltration of the posterior horn of the external meniscus, which is conducted from within in order to avoid vascular/nerve damage (Figure 4).

This technique is justified owing to the high density of the meniscus compared with other tissues like muscle or tendon, and a high pressure is required to spread the PRP into meniscus. The meniscal wall should be maintained whenever possible in order to reach a partial repair and healing process, since it is the area where the cells and blood vessels are to be found and it will induce the biological elements required for regeneration. Finally, an intra-articular infiltration is performed with 8 mL of activated F2 (Figure 4).

### 4.2. Meniscal Sutures

When feasible, meniscal sutures allow reconstituting the fine anatomy of the joint and achieving greater stability and protection of cartilage. In this case PRGF-Endoret infiltration will be conducted into the suture area and meniscal wall, and when the whole process is finished, the knee will be infiltrated in an intra-articular way. The infiltration protocol is the same as that explained in the meniscectomies (Figure 4).

Depending on the patient's progress, an outpatient intra-articular infiltration after two weeks can be taken into consideration, in order to enhance recovery.

## 5. PRP and Chondral Surgery

The treatment of cartilage injuries remains daunting despite both advances in pharmacological management of the pain and inflammation and advances in the surgical procedures and techniques. The application of PRP intra-articular injections is underpinned by a substantial body of evidence in basic science, as well as in preclinical and clinical levels of practice [51]. With this biological approach, new perspectives in knee surgery have been opened, and, drawing on the aforementioned evidence, we led to suggest four synergetic effects of PRGF-Endoret on cartilage diseases [51]. The first one is a chondroprotective effect from both the hyaluronic acid secretion by synoviocytes and the arresting of type II collagen, cleavage by the combination of TGF*β* and FGF [38, 39]. Second, an anti-inflammatory effect on human chondrocytes on the basis of the HGF effect present in PRP as well as secreted by the synoviocytes inhibits the intracellular signaling regulator of the inflammatory pathway NF-k*β* [32, 40]. The third is a cell-phenotypic modulation of chondrocytes, preventing hypertrophic differentiation and maintaining them in an arrested state, and MSCs, which promote chondrogenic differentiation. They migrate from vascular areas (synovium and subchondral bone) towards injured areas under the action of PRP and growth factors such as TGF *β*, IGFs, or FGF-2. Fourth, by attenuating and reducing joint pain, physical activity levels might improve and increase the physiological load tolerable for the joints. The increased tolerable physical load might entail a chondroprotective effect, since it has been proved that moderate mechanical loading has an anticatabolic effect on the articular cartilage either through the action of CITED2 or by suppressing NF-k*β* activation [52].

All procedures described below are based on a fracture/avulsion case published by our group. We observed how the integration of a cartilaginous fragment by arthroscopy combined with the use of PRGF-Endoret achieved an excellent cartilage repair [5].

### 5.1. Fracture/Avulsion and Osteochondritis Dissecans

Firstly, the osteochondral wound bed is debrided and the fragment separated; the bone surface of the fragment is refreshed in order to reach bone with a suitable appearance. Next, a bleeding bed is achieved by spongialization, and 3 mL of activated F2 is infiltrated into the wound bed in an intraosseous manner. After fixing the osteochondral fragment into its original niche and ensuring its stability, 2 mL of activated F2 will be infiltrated again using a fine needle. This infiltration is applied into space between the crater and the fragment; thus, the area around all edges of the reinserted fragment is filled and sealed (Figure 3).

### 5.2. Osteochondral Injuries with an Inviable Fragment

As in the previous case, the subchondral bone is debrided and all damaged tissue is removed. Again, a bleeding bed is achieved by spongialization and bone is drilled by using the Pridie procedure or microfractures. Next, it is infiltrated with liquid activated F2 by means of a trocar specially designed for this arthroscopic application (Figure 3). With this step, multipotent mesenchymal stem cells are mobilized, and generated cell signals (SDF-1 and other chemokines) trigger the joint cartilage repair process. The cells migrating to the area of the lesion will be trapped in a three-dimensional fibrin scaffold formed from PRGF-Endoret. This fact contributes to the synthesis of new tissue which performs the same mechanical function as the original.

### 5.3. Extensive Osteochondral Injuries and Necrosis

After debridement of the injured tissue and until a bleeding spongy bone bed is achieved, a series of microfractures is performed and the intra-articular wash serum is aspirated. Next, two infiltrations of activated liquid F2 are performed: intraosseous (3–5 mL) and intra-articular (8 mL) (Figure 3).

In cases like those involving the internal condyle of the knee, where there is osteonecrosis with severe involvement of the subchondral bone, autologous osteochondral grafts are performed. Depending on the size of the injury, large-scale osteochondral grafts with a fresh frozen allograft can be used by means of an open-sky surgical technique.

The integration is improved by infiltrated liquid activated F2 into the bed and bony part of the osteochondral graft. An additional infiltration is performed in the interface when the allograft has been inserted (Figure 3).

In all the cases described here, the last step of surgery consists in the aspiration of serum and as many intra-articular washes as possible and in an intra-articular infiltration of liquid activated F2. A further three intra-articular infiltrations of 8 mL on a weekly basis are conducted during the postoperative period, on an outpatient basis (Figure 3). During the first four weeks postintervention chondrocyte synthesis has to be stimulated and an anabolic environment has to be promoted. Therefore, assisted walking with crutches and a minimal initial load is recommended. Two weeks after surgery, rehabilitation should involve passive mobility and avoidance of axial movements. After week 4, partial support and resistance-free cycling together with swimming pool exercises are encouraged.

## 6. Concluding Remarks

There is a great deal of research demonstrating the safety and efficacy of PRPs in the field of orthopedic surgery. Drawing on biological evidence, our team has developed several innovative procedures for the arthroscopic repair of knee injuries assisted by PRGF-Endoret. Collectively the application of tissue-engineering biology to repair and reconstruct anatomical parts of the knee, using different formulations of PRGF-Endoret, has yielded promising clinical outcomes. These efforts point to a future where tailored PRPs will be used for each specific medical purpose.
